# People over 65 Years Old in Social Isolation: Description of an Effective Community Intervention in the City of Madrid (Spain)

**DOI:** 10.3390/ijerph19052665

**Published:** 2022-02-25

**Authors:** Ana Belén Santos-Olmo, Berta Ausín, Manuel Muñoz

**Affiliations:** School of Psychology, Personality, Evaluation and Clinical Psychology Department, Complutense University of Madrid, Campus de Somosaguas, Ctra. de Húmera, s/n, Pozuelo de Alarcón, 28223 Madrid, Spain; anabsant@ucm.es (A.B.S.-O.); mmunozlo@ucm.es (M.M.)

**Keywords:** social isolation, loneliness, older adults, intervention, mental health

## Abstract

Loneliness and social isolation in the elderly population can be shown to be a significant independent risk factor for several conditions, such as poor health behaviours, physical health problems and psychiatric conditions. Although, in the last 20 years, several interventions have been developed to reduce the impact of social isolation and loneliness on the health of older people. However, only a small proportion of these interventions are effective. This study aims to describe the components of the Psychological Support Service for Socially Isolated Elderly People (PSIE), in addition to analysing the effectiveness of a community intervention based on an outreach strategy to combat situations of social isolation in the elderly population. The sample consisted of 63 people over 65 years of age from the city of Madrid (Spain), detected by the socio-health services as people at risk of social isolation. Sociodemographic, mental health, health and psychosocial functioning, global functioning, disability and socio-sanitary needs were evaluated with observational scales. Descriptive statistics were calculated for sociodemographic and mental health variables. An analysis was carried out to study the possible influence of gender in the initial sample on the different variables assessed, using Chi-squared and Student’s *t*-tests for independent samples, with measures of effect size in each case. A study of the effectiveness of PSIE was carried out with an analysis of pre- and post-treatment measures. A Student’s *t*-test was used for related samples, as well as the effect size of Cohen’s d statistic. For the assessment of the possible influence of gender on the results of the intervention, a 2 × 2 repeated-measures ANOVA (pre-/post-measures × gender) was conducted. Regarding mental health, 65.2% of the sample presented symptoms compatible with a severe mental disorder, the most frequent being psychotic disorder (22.7%), alcohol use disorder (16.7%), personality disorder (15.2%), anxiety disorders (10.4%) and mood disorders (10.4%). The gender variable does not seem to have an influence on any of the outcome measures studied. The results of the effectiveness study indicate that the PSIE is an intervention programme that serves to improve the scores of people in the sample in all variables that the programme studied. Some of the components of PSIE that could explain its effectiveness are individualized interventions, with a home-based approach by professionals, serving as a link between the older person and the normalized social-sanitary network. Further research is required to provide more robust data on the effectiveness of interventions.

## 1. Introduction

Isolation has been defined in various ways in the scientific literature and is often confused with loneliness and exclusion. It has been defined as the objective measure of having minimal interactions with others (social isolation) and as the subjective feeling of dissatisfaction with the low number of social contacts maintained (emotional isolation or loneliness) [1]. Social isolation has also been defined as living without companionship, social support or social connection [2]; as a state in which a person or group feels the need or desire to become more involved with others but is unable to establish that contact [3]; and as a state in which the individual lacks a sense of social belonging and bonding or commitment to others and has few social contacts that do not constitute quality relationships [4]. Along the same lines, loneliness or emotional isolation has been defined as the (subjective) feeling of lack or loss of companionship, while they consider that social isolation is the (objective) absence of contacts and interactions between an older person and their social network [5]. The parallelism between “objective loneliness- social loneliness- social isolation” and “subjective loneliness- emotional loneliness- emotional isolation” is obvious and, for many authors, shapes the meaning of both terms. In general, social isolation refers both to the absence of satisfactory social relationships and to a low level of participation in community life.

Several studies analysed the relationship between social isolation and loneliness on the health of the population. The review conducted by Mushtaq et al. [6] found that loneliness and social isolation can lead to various psychiatric disorders such as depression, alcohol use disorder, child abuse, sleep problems, personality disorders and Alzheimer’s disease. Other authors have found that having social support is related to a lower perception of pain [7] and experiencing a greater life satisfaction [8,9]. It should be added that the health and well-being consequences of social isolation and loneliness in old age are increasingly being recognized. Courtin and Knapp [10] searched nine databases for empirical papers investigating the impact of social isolation and/or loneliness on a range of health outcomes in old age (128 items from 15 countries were included in the scoping review). These authors found that depression and cardiovascular health are the most often-researched outcomes, followed by well-being. Almost all studies found a detrimental effect of isolation or loneliness on health. However, causal links and mechanisms are difficult to demonstrate, and these authors suggest that further investigation is warranted. They found a paucity of research focusing on at-risk sub-groups and in the area of interventions. This also leads to various physical disorders such as diabetes; autoimmune disorders such as rheumatoid arthritis and lupus; and cardiovascular diseases such as coronary heart disease, hypertension, obesity, physiological ageing, cancer, poor hearing and poor health. In addition, the review by Crewdson [11] indicates that loneliness and social isolation in the elderly population is shown to be a significant independent risk factor for several conditions such as poor health behaviours (e.g., smoking and alcohol consumption), physical health problems (e.g., hypertension and motor decline), and psychiatric conditions (e.g., depression and cognitive impairment). In addition to this, a systematic review and meta-analysis of longitudinal studies [12] report that loneliness was positively associated with an increased risk of dementia; while older people’s sense of identity helped them to compensate and adapt to the negative effects of the well-being of memory loss [13].

In the last 20 years, several interventions were developed to reduce the impact of social isolation and loneliness on the health of older people. The effectiveness of these interventions has been analysed in various systematic reviews [5,14,15,16,17,18,19,20,21,22]. Among the conclusions of these reviews are that only a small proportion of these interventions are effective. It is observed that the interventions that demonstrated some effectiveness are those that include some educational component, social or group activities, well-trained staff facilitating social activity or support and those that are aimed at a specific population group (e.g., widowers, women, etc.). In addition, when thinking about interventions with people in social isolation, who are unlikely to attend a senior centre voluntarily, it is important to highlight the more individualized interventions, with a home approach by a professional, to assess the health and needs of the elderly person in social isolation and to serve as a link between the elderly person and the network of normalized social and health resources. In addition, interventions seem to be more effective if they include more than one methodology. In this same sense, outreach processes are not sufficient as the only element of the intervention but can be very useful if they are combined with engagement and intervention strategies. In addition, the high-quality selection, training and support for intervention facilitators and coordinators is one of the most important factors underpinning successful interventions. On the contrary, two factors are noted that make interventions more likely to be successful. On the one hand, interventions are more effective if the older person is involved in all steps: the planning, implementation and evaluation of the intervention. On the other hand, interventions are more likely to be successful if they use community resources and provide the community with tools and the capacity to help.

Fakoya [21] points out that the individuality of the experience of loneliness and isolation may cause difficulty in the delivery of standardized interventions. In this regard, there is no one-size-fits-all approach to addressing loneliness or social isolation, hence the need to tailor interventions to suit the needs of individuals, specific groups or the degree of loneliness experienced. Therefore, future research should aim to discern which intervention works for whom, in what particular context and how. Furthermore, the quality of the evidence base is weak and further research is required to provide more robust data on the effectiveness of interventions [20].

Based on the above evidence, it is necessary to design and implement interventions at the community level to reduce the loneliness and social isolation of older people, to order to minimize the negative effects they can have on the health of this population. This study aims to describe the components of the Psychological Support Service for Socially Isolated Elderly People (PSIE), as well as analysing the effectiveness of a community intervention based on an outreach strategy to combat situations of social isolation in the elderly population of the city of Madrid (Spain).

## 2. Materials and Methods

### 2.1. Design and Procedure for the Application of the Psychological Support Service for Socially Isolated Elderly People (PSIE)

To design this service, on the one hand, an extensive search of bibliographic information was carried out with different sources, trying to locate research projects or services in operation at a public or private level whose object of intervention was elderly people in social isolation; on the contrary, a qualitative study was carried out through groups of experts in the area of social exclusion, the elderly and other vulnerable groups (for example, homeless people and severe mental illness). The result of this work was the proposal of a psychological home care service for elderly people in social isolation. This research aims to present the effectiveness of the data after their implementation. This service is based on 5 basic principles: quality, outreach, case management, personal assistance and continuity of care, and it is composed of three protocols: contact and engagement, evaluation and intervention. In this case, it is a psychological care service integrated within the municipal care network of Madrid City Council.

The general objective of the PSIE is to achieve an approach to frail elderly people with a clear risk of social isolation that guides their situations towards inclusion and normalization, assigning the appropriate social resources in each case, or as a last resort, supporting involuntary institutionalization and/or legal incapacitation. The specific objectives are the following:

1. To carry out an engagement work (through the outreach strategy) on people in social isolation, both with the PSIE and with the normalized social-sanitary network.
2. To carry out an assessment that is as exhaustive as possible depending on the case, including data on health assessment, psychosocial functioning, level of disability and unmet needs.
3. To carry out a psychosocial intervention to cover the person’s needs through a process of inclusion and normalization or, as a last resort, to support the process of non-voluntary institutionalization and/or legal incapacitation.
4. Strengthen the social network of the elderly person in isolation, working with potential informal support agents in the process of contact/engagement, assessment and intervention.
5. Destigmatize the person in isolation, both in their community environment and in their family context.

The three PSIE action protocols are described below:

(1) Contact and engagement protocol

The strategy for approaching the elderly person is through home visits, carried out by PSIE psychologists alone or accompanied by facilitating agents to establish the therapeutic relationship (professional facilitating agents or from the social and family network). The system of appointments in a centre is not used, but instead, the professionals of the street team contact the person in the community. Contact is always made in a person’s own environment, preferably in their home, and if it is impossible to contact them in their home, attempts are made in the places in the community that they frequent, with total respect and care for data protection and the person’s privacy. Non-face-to-face contact can be carried out via emails, phone calls or by sending letters. The frequency of contacts depends on the response of the elderly person to the presence of the team; however, there will always be a minimum of one contact per month, either personal or non-face-to-face. The objective is to contact the person regularly to help him or she get used to the team’s presence and gradually build trust. The presence of other professionals during PSIE visits is assessed according to the intervention needs and the degree of trust and engagement with the elderly person.

(2) Assessment Protocol

Once an initial level of engagement has been achieved, the psychological and social assessment of the new case begins in parallel. A clinical (physical health); functional (global functioning and disability); psychological, cognitive and social functioning; and needs assessment are performed.

For this purpose, the following assessment instruments are used and will be administered in all cases:

- Home Health Outcome Scales for People Over 65 (HoNOS65+) [23] in its Spanish adaptation [24].
- Global Assessment of Functioning (GAF) [25] in its Spanish adaptation [26].
- WHO Brief Disability Assessment Scale (WHO-DAS-S) [27] in its Spanish adaptation [28].
- Camberwell Needs Assessment Questionnaire for the Elderly (CANE) [29] in its Spanish adaptation [30].
- Other assessment scales can be added to this assessment as considered appropriate according to the specific case.

Given the characteristics of the sample regarding the rejection of previous interventions and reluctance to the presence of professionals, none of the selected instruments will be completed by the person being assessed; instead, they will be assessed by the PSIE professionals based on the information gathered through direct observation and interview, both from the user and from other sources of information (professional facilitators or from the social and family networks of the person being assessed). In this way, the process of assessment of this population is simplified.

(3) Intervention Protocol

During the intervention, the main objective of the PSIE is not to directly treat the social or mental health problems that the person may present; instead, the priority approach is to normalize the care and connect or “reconnect” the person with normalized social and health networks, where a solution can be given to those social or health problems that the person may present. In other words, the PSIE facilitates contact between the elderly person and social and health services (both primary and specialized care) in their area, so that they can receive the care they need on each occasion. In these cases, the work of PSIE focuses on the detection of problems, is a social and psychological accompaniment for the beginning and continuity of the necessary treatments and interventions in each case, and helps to change the attitude of the elderly person in social isolation, encouraging them to accept the help they need, which they previously rejected. This research of awareness, contact with elderly people’s realities and the acceptance of resources is conducted globally through psychological intervention strategies:

- Intervention in the clinical area (physical health) and psychological and cognitive functioning: The objective is the acceptance and engagement of the person (or in some cases, maintenance) with normalized health services, both at primary level (primary health) and specialized level (especially neurology, geriatrics and mental health: Psychiatry/psychology).
- Intervention in global functioning and disability: The objective is the acceptance and engagement of the person in those resources that assist in the activities of daily living, both basic and instrumental (home help service, home meal service, laundry service, etc.). These services are usually provided by social services, but may also come from other organizations, such as religious or voluntary organizations, etc.
- Intervention in social functioning: The objective is to create, improve and/or maintain the person’s social support network, both at the informal and formal level, favouring the person’s approach to family and friends or creating new social networks through volunteering, municipal senior centres or community associations; working on neighbourhood support and the stigma of the person in social isolation in the community; and promoting leisure activities, etc.
- Intervention in the area of unmet needs: The objective is to cover the unmet needs of the person. This area is transversal to the areas mentioned above.

For the intervention, support will be provided by professional facilitators and the social and family network of the person in social isolation whenever possible.

PSIE psychologists participate in training sessions on social isolation in the elderly for professionals of the social–health network, as well as specific training for professionals involved in specific cases, as well as family members, friends or other members of the support network of the elderly person in social isolation.

In those cases, in which the level of deterioration is irrecoverable or there is an imminent risk to the health of the person or that of others, the opening of a file in the Office of the Public Prosecutor for the Elderly will be supported to assess the advisability of non-voluntary institutionalization in a hospital or residential centre and/or legal incapacitation as a protective measure in a situation of clear lack of protection and risk.

Once the intervention has been completed, the PSIE follows up on all cases closed within three to six months after closure. The follow-up consists of retrieving information on the persons served through contact with the user, the professionals involved (mental health, social services, residential centre, etc.) and/or the person’s informal network (family members, caregivers, etc.). To elaborate on this follow-up, the same measures are used as in the assessment protocol. If the person has returned to a situation of social isolation, the case is reopened by the social services. On the other hand, if the situation remains non-isolated, the PSIE closes the case definitively, leaving the follow-up to the municipal social services.

### 2.2. Study of the Effectiveness of the Psychological Support Service for Socially Isolated Elderly People (PSIE)

#### 2.2.1. Participants and Procedure

Cases are detected and referred to PSIE through the General Directorate for the Elderly and the Social Services of the Madrid City Council (Spain). For the PSIE to begin its intervention, the case must meet certain inclusion criteria: be 65 years of age or older; live alone or live with other people over 65; have uncovered social and/or health needs; have little or no social support network; refuse the assistance offered to cover their needs from the normalized social and/or health services; municipal social services have intervened, without having resolved the situation of social isolation, and consider that all avenues of intervention have been exhausted.

The intervention was carried out with all the people who were detected and referred to the PSIE, so the sample is incidental.

The sample of the effectiveness study consists of 68 people over 65 in social isolation in the city of Madrid (Spain), in which the PSIE carried out the complete intervention, until its closure, which allowed for the collection of pre- and post-treatment data. All participants in whom at least one post-treatment measurement could be taken were considered for analysis.

Regarding the procedure carried out, the pre-measures were taken at the initial stages of the intervention, reflecting the situation of the elderly person at the time of referral to the PSIE; while the post-measures were taken 3 to 6 months after the end of the PSIE intervention. Given that the conditions of the sample are of great deterioration and chronicity in their state of isolation, the post-treatment measures are delayed until a few months after the end of the intervention to assess not only the achievement of the intervention objectives but also their consolidation in the medium term.

For ethical reasons, it was not possible to have a control group, since, given the seriousness of the situation of the cases of elderly people in social isolation detected and referred to the PSIE, the intervention was started for all of them.

Informed consent was requested from the people in the sample. The study was conducted following the Declaration of Helsinki, and the protocol was approved by the Department of Programming, Evaluation and Development of the General Directorate for the Elderly of the Madrid City Council, code 001/085/233.01/489.01.

#### 2.2.2. Measures

The following instruments were included in the assessment:

− Health and Psychosocial Functioning, through the use of the Spanish adaptation of the Health Outcome Scales for People Over 65 (HoNOS65+) [24]: An outcome assessment instrument for the elderly population consisting of 12 scales that assess various physical and psychosocial problems, which are scored in a range of severity from 0 (no problem), 1 (minor problem), 2 (mild problem), 3 (moderate problem) to 4 (severe problem), with a total sum score added. Scores range from 0–48 points, with higher scores indicating poorer health and functioning. The Spanish adaptation of the scale [24] has adequate psychometric criteria (statistically significant concurrent validity values ranging from 0.23 to 0.89; and inter-rater reliability values close to 1).
− Global functioning, using the Spanish version of the Global Assessment of Functioning (GAF) [26]. This scale, included in Axis V of the DSM-IV-TR diagnostic manual (American Psychiatric Association, 2000), measures the psychosocial functioning of the individual, through the assessment of the severity of symptoms and activity impairment. It is designed for the general population. It is a 100-item scale, in which scores close to 0 indicate poorer functioning. It has good psychometric properties with an intraclass correlation coefficient of 0.57.
− Disability, using the Spanish adaptation of the WHO Disability Assessment Short Scale (WHO-DAS-S) [28]. The short version of the semi-structured interview WHO-Psychiatric Disability Assessment Schedule (WHO-DAS). It consists of 4 areas to be assessed by the clinician during the interview. Based on the information obtained, the clinician must score the person’s impairment on a visual analogue scale from 0 (“no disability”) to 5 (“maximum disability”) according to defined criteria. It has good psychometric properties, with an intraclass correlation coefficient ranging from 0.40 (for the family/housing category) to 0.74 (personal care), so that half of the specified disability categories have kappa values above 0.50, and the other half between 0.40 and 0.50.
− Social health needs, using Section 1 of the Spanish adaptation of the Camberwell Needs Assessment Questionnaire for the Elderly (CANE) [30]: This instrument is specifically designed to measure the needs of people with a possible mental disorder over 65 years of age. Section 1 (whether there is a current problem in any of the areas assessed) was used, which consists of 24 areas of assessment relating to the user/patient, plus another 2 (A and B) that refer to the main caregiver but have not been included in the present study given the special characteristics of isolation of the sample and the lack of a main caregiver. Each of these 24 areas is scored: 0 (no serious problem); 1 (there is a problem, but it is covered due to the help provided); 2 (a serious problem is present and not covered, despite any help being provided). The Spanish version of the scale has a good concurrent validity, high test–retest reliability (kappa between 0.65 and 1) and high inter-rater reliability (kappa between 0.60 and 1) [30].

#### 2.2.3. Data Analysis

Descriptive statistics were calculated for socio-demographic and psychological variables (mental health, cognitive impairment, health and psychosocial functioning, global assessment of functioning, disability and social health needs). An analysis was carried out to study the possible influence of gender in the initial sample on the different variables assessed, using Chi-squared and Student’s *t*-tests for independent samples, with measures of effect size in each case. A study of the effectiveness of PSIE was carried out with an analysis of pre-and post-treatment measures. Student’s t statistic for related samples was used, as well as the effect size of Cohen’s d statistic. For the assessment of the possible influence of gender on the results of the intervention, a 2 × 2 repeated measures ANOVA (pre-/post-measures × gender) was carried out. All analyses were conducted using SPSS v. 22.0 (IBM, Armonk, NY, USA) [31].

## 3. Results

### 3.1. Sociodemographic Data of the Sample

The sample (*N* = 68) contained a majority of women (73.5%), and the mean age was 81.8 years (MIN = 66, MAX = 97, SD = 6.9). The majority of the sample (86.8%) did not have a partner, 72.1% lived alone and 79.4% did not have children (Table 1).

### 3.2. Description of the Sample in Terms of Psychological Variables: Mental Health, Cognitive Impairment, Health and Psychosocial Functioning, Global Assessment of Functioning, Disability and Social Health Needs

Regarding mental health, 65.2% of the sample presented symptoms compatible with a severe mental disorder, the most frequent being psychotic disorder (22.7%), alcohol use disorder (16.7%), personality disorder (15.2%), anxiety disorders (10.4%) and mood disorders (10.4%). In 66.7% of the cases, this mental health disorder had not been detected before the PSIE intervention and only 4.8% were receiving some type of mental health treatment. Forty-seven per cent of the sample presented moderate (37.9%)-to-severe (9.1%) cognitive impairment. This level of serious deterioration concerning the mental health of the sample is reflected in the fact that 20.6% of the persons attended by the PSIE already had an open judicial file requesting protection and custody measures at the time of referral (Table 2).

Concerning health and psychosocial functioning, (HoNOS65+ scales), it can be seen that the scales where the lowest scores are found are those related to depressive disorders, psychotic disorders and suicide risk. On the other hand, the sample presents moderate-to-severe difficulties on scales 9, 10, 11 and 12, related to constructive leisure and social support, activities of daily living and living conditions. This result is supported by the mean score on the GAF instrument close to 50, indicating any severe impairment in social or occupational activity. Scale 5, related to physical health problems and disability, also shows a high mean, indicating relevant problems in this area. These data are corroborated in the disability scale WHO-DAS-S, where the difficulties in the four subscales reach a mean of serious disability (Table 2).

Regarding the needs assessment, the sample shows an average unmet needs score of almost 11, which is a high level of need considering that the instrument includes such basic needs as food, medical follow-up or housing conditions (Table 2).

### 3.3. Description and Analysis of Gender Differences in Psychological Variables

As mentioned above, the percentage of men and women in the sample is very different, with a greater presence of women (73.5%). An analysis was carried out to study the possible gender differences and their relevance with respect to the initial situation of the sample. The results are shown in Table 3.

There are statistically significant differences in the five measures. First, cognitive impairment, had a higher percentage of moderate–severe impairment in women (56.2%) than in men (22.2%); however, if we look at the effect size, the relationship of this measure with the gender variable is not very strong (Phi = 0.304). Medium effect sizes are found in the other four measures: problems associated with alcohol use (scale 3, HoNOS65+) showed a mild alcohol use problem in men and was close to 0 in women; problems related to hallucinations or delusions (scale 6, HoNOS65+) showed a mild problem in women and was close to 0 in men; other mental problems (scale 8, HoNOS65+) showed mild problems in both cases, but slightly more pronounced in men; and an average level of disability in terms of personal care and survival (scale A, WHO-DAS-S) was higher in men (level close to moderate disability) than in women (level close to serious disability). In the rest of the measures evaluated, there are no statistically significant differences according to gender.

### 3.4. Duration of PSIE Interventions and Reasons for Closure

Concerning the characteristics of the interventions, they had a mean duration of 8 months (MIN = 1, MAX = 23, SD = 6.2) and required a mean of 27 contacts (MIN = 2, MAX = 127, SD = 23.1), that is, an average of 3 contacts per month.

Regarding the reasons for the closure, 63.3% of the cases were resolved after taking legal protection actions to ensure the well-being of the elderly; and in the remaining 36.7%, the situation of social isolation was redirected towards normalization through the acceptance of formal aid and/or the strengthening of the social network of the elderly.

### 3.5. Effectiveness Study

#### 3.5.1. Health and Psychosocial Functioning (HoNOS65+)

The general trend is towards maintenance or improvement in all variables of the HoNOS65+ scales (Table 4). In all of them, the mean score decreases, indicating improvement (lower scores indicate better functioning). Significant differences are found between pre- and post-treatment in 7 of the 12 scales included in the instrument. The differences are found both in scales of psychopathological symptomatology and behavioural alteration, such as scales 1, 3, 6 and 8; scales of functioning more related to social aspects, such as scales 9 and 12; and scale 11, more related to housing conditions, both in terms of hygiene and habitability, decoration, stimuli present, etc. Concerning the total score of the HoNOS65+, significant differences were also observed between pre- (21.5) and post-treatment (13.72), showing a significant improvement of the sample in this variable. Medium-to-large effect sizes were found in all scales where there were significant differences, with the largest being found in scales related to social support, leisure activities and living conditions. Differences in the HoNOS65+ total score also indicate a large effect size.

#### 3.5.2. Global Assessment of Functioning (GAF)

The general trend is towards improvement in global functioning, with a higher post-treatment mean (in this instrument, higher scores indicate better global functioning). These differences between pre- and post-treatment are statistically significant and have a medium effect size (Table 5).

#### 3.5.3. Disability (WHO-DAS-S)

The pre-/post-data in the WHO-DAS-S instrument are for 46 persons. There are significant differences and large effect sizes in the four areas of disability included in the instrument, with the trend in all of them being towards improvement, that is, towards the more adequate functioning of the persons in the sample (Table 6). In all of them, the mean score decreases, which indicates an improvement, since, in this scale, lower scores indicate lower levels of disability. For the analyses carried out with this instrument, “obvious disability” (2) to “maximum” (5) was chosen as the criterion for the highest severity, since from a score of 2 on this scale (obvious disability), interference with a person’s social adaptation begins to be considered.

#### 3.5.4. Social Health Needs (CANE)

In the 24 areas of need, the score 0 indicates that there are no needs; 1 means that the need exists but is covered; and 2 indicates that the need is not covered. The general trend is towards fewer unmet needs, decreasing from an average of 10.68 unmet needs at pre-treatment to an average of 1.44 unmet needs at post-treatment (Table 7).

#### 3.5.5. Study of the Influence of Gender on Intervention Outcomes

The gender variable does not seem to have an influence on any of the outcome measures studied, except for alcohol use. In this variable, the results indicate that a reduction in use was achieved in both men and women, but in women a total reduction in alcohol consumption was achieved. (Table 8).

## 4. Discussion

This study aimed to describe the components of the PSIE and to analyse the effectiveness of a community intervention.

The sample shows a moderate-to-severe level of mental health deterioration, with high percentages of symptoms compatible with at least one severe mental disorder, including psychotic symptoms, alcohol use disorder, personality disorders, anxiety and mood disorders; the high presence of moderate and severe cognitive impairment; and even judicial records requesting protection measures before a referral to PSIE. Given the characteristics of the sample of isolation and rejection of social and health resources, in most cases, they are not receiving any mental health treatment. The data highlight the need for mental health care in this population. In addition, the people in the sample of this study had a very high mean score of unmet social and health needs before the intervention (10.68) compared to the average in the general population (7) [32].

Concerning interventions, the duration and number of contacts required in each case will depend on the individual characteristics of each person treated, as evidenced by the variability in the minimum and maximum ranges of both the duration and number of contacts. However, the data point to interventions of a medium and long duration with frequent contacts (around an average of 3 contacts per month).

Men seem to show a tendency towards greater alcohol use and greater difficulties in self-care; while women in the sample present a worse cognitive status and a greater (although low) presence of problems with hallucinations or delusions. However, it should be noted that the results of the analysis by gender show that the differences in this variable in most of the measures are not significant and the effect is low or medium.

The results of the effectiveness study indicate that the PSIE is an intervention programme that serves to improve the scores of the people in the sample in all the variables studied. Regarding the changes in the measure of health and psychosocial functioning (HoNOS65+), the general tendency is towards maintenance or improvement in all the variables of the HoNOS65+ scales, with significant differences in 7 of the 12 subscales. It should be noted that the intervention is effective for reducing behavioural disturbance, alcohol use, psychotic symptomatology, other emotional problems such as anxiety, and depressive symptomatology, as well as improving the frequency of social contacts, a person’s living environment, and participation in leisure activities, the latter being where the intervention shows the clearest effects. These achievements were made possible because the PSIE has engaged with elderly people in a situation of social isolation and is able to put them in contact with regular health and social services. In this way, many people with mental disorders have received care from mental health services, which has led to a reduction in mental health symptoms (mainly delusions, anxiety, depression and sleep problems). On the other hand, the PSIE has facilitated the acceptance of different social resources by people in social isolation, such as the home help service for household chores, personal hygiene, shopping, medical appointments, the home meal service, the Tele assistance service, or access to a residence for the elderly. This could explain the improvement in the HoNOS65+ subscales of living conditions, occupational activities and social relations. In addition, these social relationships may also have been improved by the inclusion of volunteers in the cases of people in whom emotional loneliness was detected. On the other hand, some HoNOS65+ subscales have not experienced significant improvements, such as cognitive problems (which are difficult to improve given that in many cases there are processes of dementia), and problems of disability and functioning in activities of daily living. It seems reasonable that this last aspect would be improved by incorporating a person from the home help service. However, the home help service was accepted on many occasions for cleaning the home but not for personal hygiene. This could explain the limited improvement in this variable.

As for the more general measures of functioning (GAF) and disability (WHO-DAS-S), statistically significant changes and medium and large effect sizes were obtained, so it can be affirmed that the PSIE, being an intervention that connects the person in social isolation with regular social and health services, helps to improve general functioning in the specific areas of personal care, occupational, family and leisure functioning.

Finally, the PSIE has proven to be effective in meeting most of the 24 needs identified by the CANE, with a general trend towards fewer unmet needs, decreasing from an average of 10.68 unmet needs at the pre-intervention time to an average of 1.44 unmet needs at the post-intervention time. It should be noted that the PSIE has served as a link to cover these needs by encouraging the elderly person in a situation of social isolation to accept different services from social and health professionals, facilitating the social support of people in their neighbourhood (shopkeepers, neighbours) and resuming contact and relationships with family members.

Regarding the effect of gender on the results of the intervention, we only found an effect on the alcohol use variable. PSIE has achieved positive results in both men and women, without gender appearing to be a relevant variable in these results. Studies included in systematic reviews and meta-analyses on the effectiveness and efficacy of interventions to reduce social isolation do not examine gender as a predictor of intervention outcomes [14,15,16,17,18,19,20,21,22]. This makes it difficult to compare our results on gender differences with previous studies. Previous studies are interested in the analysis of variables such as the intervention type (face-to-face, group or individual intervention, etc.) or the variable age, as possible predictors of the effectiveness of the interventions. Future research should focus on the study of the gender variable as a predictor of the effectiveness of interventions that aim to reduce emotional loneliness and social isolation in the elderly, in order to better plan interventions to prevent situations of loneliness and social isolation.

The effectiveness of PSIE might be because it is a programme that includes many of the ingredients recommended by different authors in different meta-analyses and reviews regarding the effectiveness of interventions to reduce social isolation and loneliness [5,14,15,16,17,18,19,20,21,22]. Some of the components of PSIE that could explain its effectiveness are individualized interventions, with a home-based approach by the professional, aiming to assess the health and needs of the older person in social isolation and serving as a link between the older person and the normalized social health network of resources. In addition, PSIE has carried out high-quality selection, training and support for the facilitators and coordinators of the interventions (home helpers, social and health workers, volunteers, among others). Another factor to be taken into account in the approach to older people in social isolation is the need to involve the elderly person in all steps, from the planning, implementation and assessment of the intervention. This factor is relevant in the PSIE, always respecting the times and rhythms of change established by the people in social isolation. Finally, a decisive factor in the effectiveness of the service is the use of community resources (volunteers, neighbours, senior centres, etc.) that have served to provide tools and support the capacity of the community itself.

A strong aspect of the present study is that it has been applied to a large sample of elderly people in social isolation and that it is one of the few studies that has been applied in the environment of the elderly person and not in a social or health centre. In other words, it was possible to access people in a situation of extreme social isolation, who do not ask for help, and who refuse any service.

One of the main limitations of the study that we found was related to the lack of a control group. For ethical reasons, it was not possible to have a control group, since, given the seriousness of the situation of the cases of elderly people in social isolation detected and referred to the PSIE, the intervention was initiated in all of them.

Regarding future research directions, it is important to include efficacy studies with a control group, in addition to conducting studies with even larger samples that include sufficient numbers of men and women to perform gender-differential analyses. On the other hand, since the COVID-19 pandemic, it seems interesting to promote the study of the efficacy of remote interventions to mitigate emotional loneliness and social isolation in the elderly [17,18,33,34,35,36]. Information and communication technologies and other traditional networks can foster social support to deal with social isolation and loneliness.

Further research is required to provide more robust data on the effectiveness of interventions, including gender differences. It is interesting to further develop theoretical understandings of how successfully interventions mediate social isolation and loneliness.

## 5. Conclusions

Older people in social isolation in the city of Madrid are in most cases women living alone, without children, and with a high percentage of severe mental disorders. This study was able to demonstrate the effectiveness of a psychological intervention with elderly people in social isolation with some specific components, such as individualized attention, intervention in the person’s own home and environment, the engagement of the elderly person with the normalized social and health care networks to meet their needs, and an additional intervention strategy focused on training and support for both the professionals involved in the case and the elderly person’s informal social support network.

## Figures and Tables

**Table 1:** Socio-demographic characteristics of the sample.

Sociodemographic Variables	*n*	%
**Sex**		
Male	18	26.5
Female	50	73.5
**Marital Status**		
Married	9	13.2
Single	35	51.5
Widowed	21	30.9
Separated or divorced	3	4.4
**Children**		
With children	12	17.6
With deceased children	2	2.9
No children	54	79.4
**Cohabiting**		
Alone	49	72.1
Spouse	9	13.2
Child	3	4.4
Sibling	7	10.3

**Table 2:** Presence of symptomatology compatible with a severe mental disorder or cognitive impairment; mental health treatment; health and psychosocial functioning, global assessment of functioning, disability and socio-sanitary needs.

Psychological Variables	*n*	%
**Presence of Severe Mental Disorder (Yes)**		
Alcohol use disorder	11	16.7
Mood disorders	7	10.4
Psychotic disorders	15	22.7
Anxiety disorders	7	10.4
Personality disorders	10	15.2
Intellectual development disorder	3	4.4
Any mental disorder (if any of the above are present)	43	65.2
**Cognitive impairment**		
No impairment	22	32.3
Mild	13	19.7
Moderate	25	37.9
Severe	6	9.1
**Mental health diagnosis and treatment before PSIE intervention**		
Detected with treatment	2	4.8
Detected without treatment	12	28.6
Not detected without treatment	28	66.7
	**Mean**	**SD**
**HoNOS65+ (Scores 0–4)**		
Scale 1. Behavioural disturbance	1.68	1.34
Scale 2. Non-accidental self-injury	0.09	0.38
Scale 3. Problem drinking or drug use	0.52	1.17
Scale 4. Cognitive problems	1.85	1.34
Scale 5. Problems related to physical illness/disability	2.6	1.1
Scale 6. Problems associated with hallucinations and/or delusions (or false beliefs)	1.01	1.52
Scale 7. Problems with depressive symptoms	0.33	0.75
Scale 8. Other mental and behavioural problems	1.08	1.35
Scale 9. Problems with social or supportive relationships	3.63	0.71
Scale 10. Problems with activities of daily living	2.94	0.86
Scale 11. Overall problems with living conditions	2.74	1.15
Scale 12. Problems with work and leisure activities/quality of daytime environment	3.6	0.67
Total HoNOS65+	21.97	4.64
**GAF (Scores 0–100)**	42.06	13
**WHO-DAS-S (Scores 0–5)**		
A. Personal care and survival	3	1.15
B. Occupational functioning	3.34	1.24
C. Family functioning	3.31	1.26
D. Functioning in other roles and leisure activities	3.26	0.75
**CANE (Scores 0–24)**		
Average number of unmet needs	10.68	3.31

**Table 3:** Description and analysis of gender differences in psychological variables.

Gender Differences in Psychological Variables	MEN (*N* = 18)		WOMEN (*N* = 50)			
	*n*	%	*n*	%	χ^2^	Phi
**Presence of any severe mental disorder**					0.178	0.052
Yes	11	61.1	32	66.7		
No	7	38.9	16	33.3		
**Cognitive impairment**					6.085 *	0.304
No impairment–mild	14	77.8	21	43.8		
Moderate–severe	4	22.2	27	56.2		
	**Mean**	**SD**	**Mean**	**SD**	**t**	**Cohen’s d**
**HoNOS65+**						
Scale 1. Behavioural disturbance	1.44	1.54	1.76	1.27	−0.85	−0.23
Scale 2. Non-accidental self-injury	0	0	0.12	0.44	−1.16	−0.32
Scale 3. Problem drinking or drug use	1.11	1.61	0.29	0.87	2.66 **	0.7
Scale 4. Cognitive problems	1.44	1.20	2	1.37	−1.6	−0.41
Scale 5. Problems related to physical illness/disability	2.5	1.10	2.64	1.10	−0.46	−0.13
Scale 6. Problems associated with hallucinations and/or delusions (or false beliefs)	0.28	0.83	1.29	1.63	−2.5 *	−0.66
Scale 7. Problems with depressive symptoms	0.17	0.51	0.40	0.82	−1.12	−0.31
Scale 8. Other mental and behavioural problems	1.67	1.50	0.85	1.24	2.06 *	0.6
Scale 9. Problems with social or supportive relationships	3.67	0.49	3.62	0.78	0.29	0.07
Scale 10. Problems with activities of daily living	3.06	0.73	2.9	0.91	0.72	0.18
Scale 11. Overall problems with living conditions	3.06	0.64	2.62	1.28	1.38	0.38
Scale 12. Problems with work and leisure activities/quality of daytime environment	3.61	0.78	3.6	0.64	0.06	0.02
Total HoNOS65+	22	5.04	21.96	4.54	0.03	0.01
**GAF**	43.06	11.26	41.7	13.65	0.41	0.1
**WHO-DAS-S**						
A. Personal care and survival	3.5	0.62	2.82	1.24	2.22 *	0.59
B. Occupational functioning	3.44	0.78	3.3	1.37	0.42	0.12
C. Family functioning	3.29	1.44	3.32	1.21	−0.07	−0.02
D. Functioning in other roles and leisure activities	3.33	0.77	3.24	0.74	0.45	0.12
**CANE**						
Average number of unmet needs	11.44	2.90	10.4	3.43	1.25	0.32

* *p* < 0.05, ** *p* < 0.01. Phi: Values close to 1 indicate larger effect sizes and stronger relationships between variables. Cohen’s d > 0.2 low effect size; >0.5 medium effect size; >0.8 large effect size.

**Table 4:** HoNOS65+ scales. Difference in the pre-/post-intervention means.

Health and Psychosocial Functioning	PRE		POST			
HoNOS65+ (*N* = 46)	Mean	SD	Mean	SD	t	Cohen’s d
Scale 1. Behavioural disturbance	1.64	1.23	0.91	0.99	4.67 ***	0.7
Scale 2. Non-accidental self-injury	0.07	0.33	0	0	1.35	0.2
Scale 3. Problem drinking or drug use	0.5	1.19	0.11	0.43	2.78 **	0.41
Scale 4. Cognitive problems	1.76	1.39	1.72	1.39	0.63	0.09
Scale 5. Problems related to physical illness/disability	2.59	1.13	2.54	1.15	0.47	0.07
Scale 6. Problems associated with hallucinations and/or delusions (or false beliefs)	0.91	1.43	0.49	0.99	3.38 **	0.5
Scale 7. Problems with depressive symptoms	0.31	0.70	0.24	0.57	1.4	0.17
Scale 8. Other mental and behavioural problems	1.09	1.34	0.2	0.76	4.65 ***	0.69
Scale 9. Problems with social or supportive relationships	3.53	0.82	2.09	1.15	8.36 ***	1.25
Scale 10. Problems with activities of daily living	2.96	0.88	2.93	0.92	0.18	0.03
Scale 11. Overall problems with living conditions	2.57	1.21	0.98	0.75	8.33 ***	1.23
Scale 12. Problems with work and leisure activities/quality of the daytime environment	3.53	0.73	2.04	1.15	8.33 ***	1.24
Total HoNOS65+	21.5	4.84	13.72	4.77	10.12 ***	1.49

** *p* < 0.01, *** *p* < 0.001. Cohen’s d > 0.2 low effect size; >0.5 medium effect size; >0.8 large effect size.

**Table 5:** GAF. Difference in the pre-/post-intervention means.

Global Assessment of Functioning	PRE		POST			
GAF (*N* = 46)	Mean	SD	Mean	SD	t	Cohen’s d
	44.67	13.18	52.24	15.95	−4.06 ***	−0.6

*** *p* < 0.001. Cohen’s d > 0.2 low effect size; >0.5 medium effect size; >0.8 large effect size.

**Table 6:** WHO-DAS-S. Difference in the pre-/post-intervention means.

Disability	PRE		POST			
WHO-DAS-S (*N* = 46)	Mean	SD	Mean	SD	t	Cohen’s d
A. Personal care and survival	2.93	1.20	1.39	0.93	8.82 ***	1.3
B. Occupational functioning	3.31	1.30	2	1.41	6.04 ***	0.93
C. Family functioning	3.12	1.19	2.03	1.67	5.42 ***	0.94
D. Functioning in other roles and leisure activities	3.23	0.75	1.95	1.11	6.98 ***	1.06

*** *p* < 0.001. Cohen’s d > 0.2 low effect size; >0.5 medium effect size; >0.8 large effect size.

**Table 7:** CANE. Difference in pre-/post-means of total unmet needs.

Social Health Needs	PRE		POST			
CANE (*N* = 68)	Mean	SD	Mean	SD	t	Cohen’s d
Average number of unmet needs	10.68	3.31	1.44	2.12	19.99 ***	2.31

*** *p* < 0.001. Cohen’s d > 0.2 low effect size; >0.5 medium effect size; >0.8 large effect size.

**Table 8:** Influence of gender on intervention outcomes.

Influence of Gender	MEN		WOMEN			
	PRE	POST	PRE	POST		
	Mean (SD)	Mean (SD)	Mean (SD)	Mean (SD)	F	dF
**HoNOS65+**						
Scale 1. Behavioural disturbance	1.46 (1.45)	0.62 (0.96)	1.72 (1.14)	1.03 (0.99)	0.206	1
Scale 2. Non-accidental self-injury	0	0	0.09 (0.39)	0	0.798	1
Scale 3. Problem drinking or drug use	1.29 (1.73)	0.36 (0.75)	0.16 (0.63)	0	7.272 **	1
Scale 4. Cognitive problems	1.21 (1.19)	1.21 (1.31)	2 (1.41)	1.94 (1.39)	0.17	1
Scale 5. Problems related to physical illness/disability	2.29 (1.14)	2.21 (1.25)	2.72 (1.11)	2.69 (1.09)	0.039	1
Scale 6. Problems associated with hallucinations and/or delusions (or false beliefs)	0.36 (0.93)	0.21 (0.8)	1.16 (1.55)	0.61 (1.05)	2.32	1
Scale 7. Problems with depressive symptoms	0.15 (0.56)	0.23 (0.6)	0.38 (0.75)	0.25 (0.57)	2.523	1
Scale 8. Other mental and behavioural problems	1.64 (1.6)	0.29 (1.07)	0.84 (1.24)	0.16 (0.58)	2.818	1
Scale 9. Problems with social or supportive relationships	3.69 (0.48)	2.15 (1.14)	3.47 (0.92)	2.06 (1.16)	0.118	1
Scale 10. Problems with activities of daily living	3.23 (0.6)	2.85 (0.8)	2.84 (0.95)	2.97 (0.97)	3.613	1
Scale 11. Overall problems with living conditions	3.07 (0.62)	1 (0.96)	2.34 (1.34)	0.97 (0.65)	2.951	1
Scale 12. Problems with work and leisure activities/quality of daytime environment	3.46 (0.88)	1.92 (1.04)	3.56 (0.67)	2.09 (1.2)	0.031	1
Total HoNOS65+	22.14 (5.68)	12.43 (5.27)	21.22 (4.49)	14.28 (4.51)	2.876	1
**GAF**	42.14 (11.22)	53.79 (16.82)	45.78 (13)	51.56 (15.78)	2.15	1
**WHO-DAS-S**						
A. Personal care and survival	3.57 (0.51)	1.79 (1.05)	2.66 (1.31)	1.22 (0.83)	0.835	1
B. Occupational functioning	3.58 (0.67)	1.83 (0.84)	3.2 (1.47)	2.07 (1.6)	1.678	1
C. Family functioning	3.3 (1.25)	1.9 (1.85)	3.04 (1.19)	2.09 (1.62)	1.027	1
D. Functioning in other roles and leisure activities	3.42 (0.67)	1.83 (1.27)	3.16 (0.78)	2 (1.07)	1.069	1
**CANE**						
Average number of unmet needs	11.44 (2.9)	1.5 (2.79)	10.4 (3.43)	1.42 (1.85)	0.768	1

** *p* < 0.01.

## Data Availability

The datasets used during the current study are available from the corresponding author on reasonable request.
